# Mucous membrane pemphigoid associated with thymoma: A rare cancer-associated immune manifestation

**DOI:** 10.1016/j.jdcr.2026.06.002

**Published:** 2026-06-12

**Authors:** Ali Fakih, Jawad Makarem, Nada Sabbah, Souad Bou Harb

**Affiliations:** a Dermatology Department, Faculty of Medical Sciences, Lebanese University, Beirut, Lebanon; b Dermatology Department, Hammoud Hospital University Medical Center, Saida, Lebanon; c Hematology Oncology Chairman of Internal Medicine Department, Ainwazein Medical Village, Mount Lebanon, Lebanon; d Hematology Oncology Department, Ainwazein Medical Village, Mount Lebanon, Lebanon; e Pathology Department, Lebanese American University, School of Medicine, Beirut, Lebanon; f Infectious Diseases Department, Mount Lebanon Hospital University Medical Center, Mount Lebanon, Lebanon; g Infectious Diseases Department, Faculty of Medicine and Medical Sciences, University of Balamand, Beirut, Lebanon

**Keywords:** autoimmune blistering disease, cancer-associated autoimmunity, immune dysregulation, mucous membrane pemphigoid, paraneoplastic syndrome, thymoma, tumor progression

## Introduction

Thymomas, the most common tumors of the anterior mediastinum, are often discovered incidentally on imaging or during evaluation for thoracic symptoms or in the context of paraneoplastic manifestations.^1^ Paraneoplastic syndromes may precede the diagnosis of thymoma, occur concurrently with the tumor, or appear after treatment, with or without evidence of recurrence. Myasthenia gravis represents the most frequent paraneoplastic condition linked to thymoma.^1^^,^^2^ Mucous membrane pemphigoid (MMP) is an uncommon autoimmune subepithelial blistering disorder that primarily affects mucosal surfaces. Although MMP is not classically considered a paraneoplastic disease, associations with underlying malignancies have been reported.^3^^,^^4^ Although thymoma is classically associated with pemphigus and paraneoplastic pemphigus, its association with mucous membrane pemphigoid is exceedingly rare.^5^ We report a rare case of mucosal erosions consistent with MMP occurring in a patient with thymoma, in which the onset of MMP coincided with objective tumor progression.

## Case

**Fig 1:**
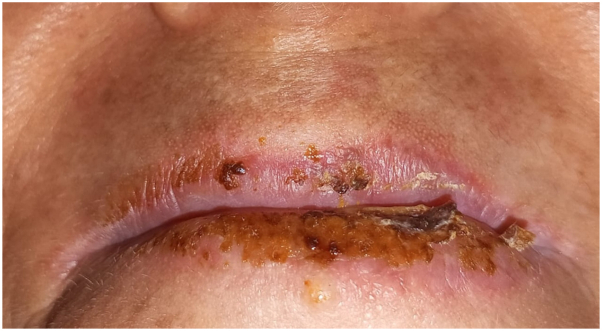
Clinical photograph of the lips showing erosions, ulcerations, and hemorrhagic crusting.

**Fig 2:**
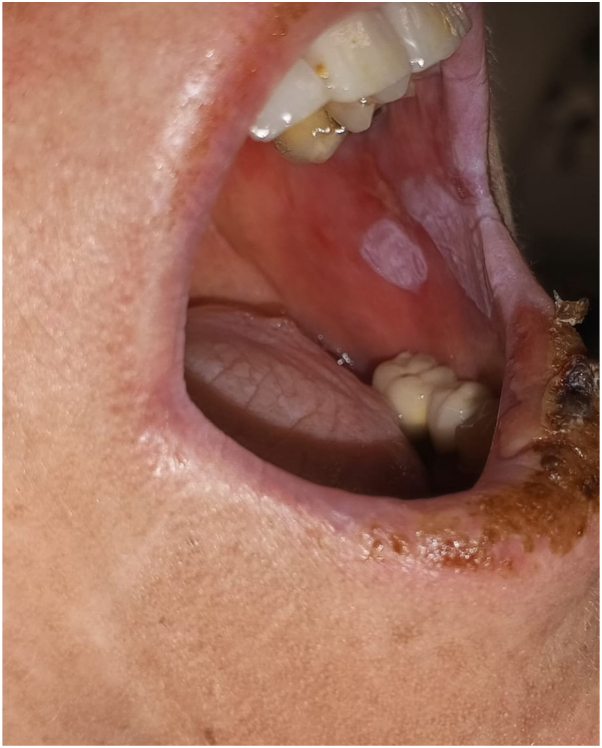
Clinical photograph of the internal oral mucosa showing whitish erosions and ulcerations unresponsive to conventional antimicrobial therapies.

**Fig 3:**
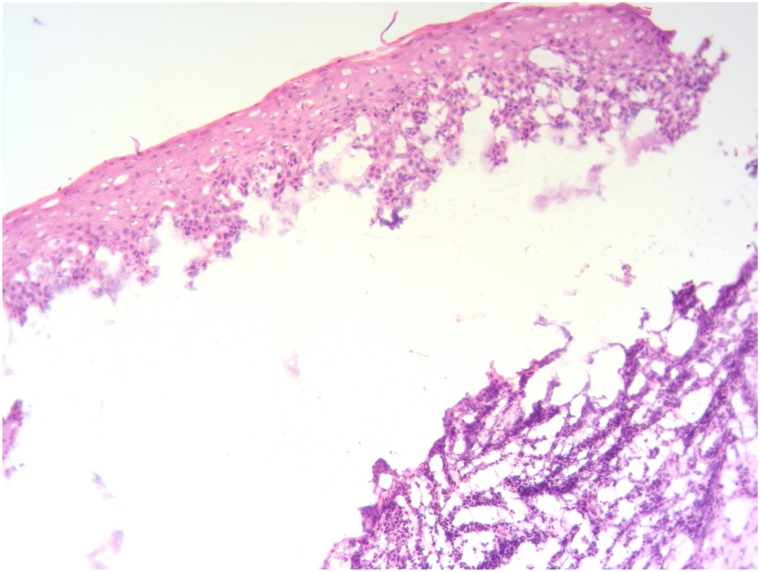
Histopathologic examination showing focal subepithelial clefting with predominantly neutrophilic inflammatory infiltrate and absence of significant eosinophils, favoring mucous membrane pemphigoid (H&E, ×100).

A 64-year-old female patient with a known type B2 thymoma, associated with mediastinal, and pleural lymphadenopathies, diagnosed in 2021, was initially treated with surgical resection. She then received adjuvant chemotherapy between 2023 and 2024. She was subsequently started on weekly carboplatin and etoposide during which painful oral ulcerations and erosions of the lips and oral mucosa appeared (Figs 1 and 2). Chemotherapy was subsequently discontinued. A positron emission tomography scan performed thereafter demonstrated stabilization of thymoma activity. She was treated empirically with oral acyclovir and oral valacyclovir without improvement. She also received oral fluconazole with no significant improvement. The patient was referred to the dermatology department for further evaluation. Physical examination revealed erosions, crusting, and scaling of the upper and lower lips, together with multiple ulcerations of the buccal mucosa. Redness and swelling of bilateral conjunctiva were also observed. No skin lesions were present. The patient additionally reported dysphagia, nasal obstruction, and painful genital lesions. Given the persistent oral erosions and the involvement of multiple mucosal sites, the differential diagnosis included viral infection, drug-induced mucositis, paraneoplastic pemphigus, bullous pemphigoid, and mucous membrane pemphigoid. The absence of response to antiviral therapy and persistence of lesions after chemotherapy discontinuation argued against infectious or toxic etiologies, prompting biopsy for histopathology and direct immunofluorescence. Histopathologic examination revealed subepithelial clefting associated with predominantly neutrophilic inflammatory infiltrate without significant eosinophils (Fig 3). No interface dermatitis or necrotic keratinocytes were identified. Focal acantholysis was observed and interpreted as secondary to epithelial damage rather than a primary acantholytic process. Direct immunofluorescence demonstrated isolated linear deposition of IgG along the basement membrane zone without intercellular staining, favoring mucous membrane pemphigoid over paraneoplastic pemphigus and bullous pemphigoid. Antilaminin 332 antibody test was not available at our institution or in the surrounding region. Clinically, the absence of cutaneous bullae, polymorphous skin lesions, respiratory involvement, and severe refractory disease further supported MMP over paraneoplastic pemphigus or bullous pemphigoid. The patient was started on oral prednisone at 0.75 mg/kg/d, with marked clinical improvement of mucosal lesions noted at 1-month follow-up. Corticosteroid therapy was subsequently tapered. During oncologic reassessment, a subsequent PET scan revealed progression of the known thymoma. The patient was referred back to the oncology unit for evaluation of further chemotherapy. Following initiation of pemetrexed, the patient reported recurrence of oral ulcerations, which again improved under systemic corticosteroid therapy at a dose of 0.3 mg/kg/d.

## Discussion

Mucous membrane pemphigoid is a rare autoimmune bullous disease, with an estimated incidence of approximately 2 cases per million inhabitants per year.^6^ MMP can be associated with internal malignancy, particularly when antilaminin 332 antibodies are positive. The pathophysiologic relationship of MMP and cancer is not well understood. However, expression of laminin 332 has been detected in malignant cells. It has been implicated in tumor cell adhesion, growth, and metastasis.^7^ In a case series of 55 patients with antilaminin 332 positive MMP, 14% had associated malignant tumors of various tissues.^3^ An increased risk of cancer in patients with other forms of MMP has also been described, although the magnitude of this risk remains uncertain.^4^ A meta-analysis of forty-seven studies grouping 1429 patients with MMP revealed that 221 of them had a history of malignancy, with breast cancer being the most commonly reported tumor type.^8^ Across these published series, none of these patients were diagnosed with thymoma. To our knowledge, 1 clearly documented case of mucous membrane pemphigoid associated with thymoma has been reported.^5^ An additional report describing a pemphigoid disorder associated with thymoma has been published; however, the exact clinical phenotype and diagnostic details were not clearly specified.^9^ In our patient, the diagnosis of MMP preceded the radiological confirmation of thymoma progression and occurred in temporal association with thymoma under chemotherapy, supporting a cancer-associated immune mechanism, possibly paraneoplastic in nature. Although thymoma is classically associated with paraneoplastic pemphigus, as demonstrated by a systematic review of published case reports,^10^ the association between MMP and thymoma is extremely rare. In our case, mucosal lesions developed during ongoing chemotherapy. While chemotherapy-induced mucositis was initially considered, the clinicopathologic findings, including subepithelial clefting and isolated linear IgG deposition along the basement membrane zone on direct immunofluorescence, favored a diagnosis of MMP. Overlap between autoimmune blistering disorders remains possible in the setting of thymoma-associated immune dysregulation. There is no evidence that etoposide or carboplatin directly induce mucous membrane pemphigoid. It is possible, therefore, that chemotherapy acted as a triggering or exacerbating factor in a patient with underlying thymoma-related immune dysregulation, rather than being the primary cause of the disease. Interestingly, mucosal lesions recurred following the initiation of pemetrexed, a chemotherapeutic agent with a different mechanism of action from etoposide and carboplatin. This relapsing course argues against a direct toxic effect of a single drug and further supports an immune-mediated process. We report a rare case of mucous membrane pemphigoid occurring in temporal association with thymoma, suggesting a probable paraneoplastic immune process. In patients presenting with new-onset mucosal ulcerations in the context of an underlying malignancy, autoimmune bullous disorders should be considered to ensure accurate diagnosis, appropriate treatment, and timely oncologic evaluation for optimal patient management.

### Declaration of generative AI and AI-assisted technologies in the writing process

During the preparation of this work, the authors used chatGPT in order to correct grammar of the article. After using this tool/service, the authors reviewed and edited the content as needed and take full responsibility for the content of the published article.

## Conflicts of interest

None disclosed.
